# Smart Materials Meet Multifunctional Biomedical Devices: Current and Prospective Implications for Nanomedicine

**DOI:** 10.3389/fbioe.2017.00080

**Published:** 2017-12-18

**Authors:** Giada Graziana Genchi, Attilio Marino, Christos Tapeinos, Gianni Ciofani

**Affiliations:** ^1^Smart Bio-Interfaces, Istituto Italiano di Tecnologia, Pontedera, Italy; ^2^Department of Mechanical and Aerospace Engineering, Politecnico di Torino, Torino, Italy

**Keywords:** smart materials, remote stimulation, drug delivery, immune system, tissue engineering

## Abstract

With the increasing advances in the fabrication and in monitoring approaches of nanotechnology devices, novel materials are being synthesized and tested for the interaction with biological environments. Among them, smart materials in particular provide versatile and dynamically tunable platforms for the investigation and manipulation of several biological activities with very low invasiveness in hardly accessible anatomical districts. In the following, we will briefly recall recent examples of nanotechnology-based materials that can be remotely activated and controlled through different sources of energy, such as electromagnetic fields or ultrasounds, for their relevance to both basic science investigations and translational nanomedicine. Moreover, we will introduce some examples of hybrid materials showing mutually beneficial components for the development of multifunctional devices, able to simultaneously perform duties like imaging, tissue targeting, drug delivery, and redox state control. Finally, we will highlight challenging perspectives for the development of theranostic agents (merging diagnostic and therapeutic functionalities), underlining open questions for these smart nanotechnology-based devices to be made readily available to the patients in need.

## Introduction

Smart materials have gained increasing attention in the biomedical research fields thanks to their adjustable physical and/or chemical properties in response to deliberately imparted external stimuli or to environmental changes. For these reasons, their introduction in nanomedicine has opened unprecedented possibilities of manipulation of biological entities at cellular and even sub-cellular level. In this scenario, the intrinsic properties of nanoparticles or nanotextured materials are exploited, providing active devices capable of diagnostic, therapeutic or even theranostic functions. When physical cues like light irradiation, ultrasounds, or electromagnetic fields are applied to a smart nanostructure, an energy transduction occurs and results into the activation of a precise cellular functionality. Moreover, a suitable modification of the nanoparticle surface (e.g., with the aid of a cell ligand or of a monoclonal antibody) can improve the efficacy of this activation, by targeting specific cell populations or even specific intracellular organelles. This approach, which can be defined as a new paradigm in nanomedicine, finds several applications including cancer therapy, drug delivery, tissue engineering, and even bionics.

**Figure 1:**
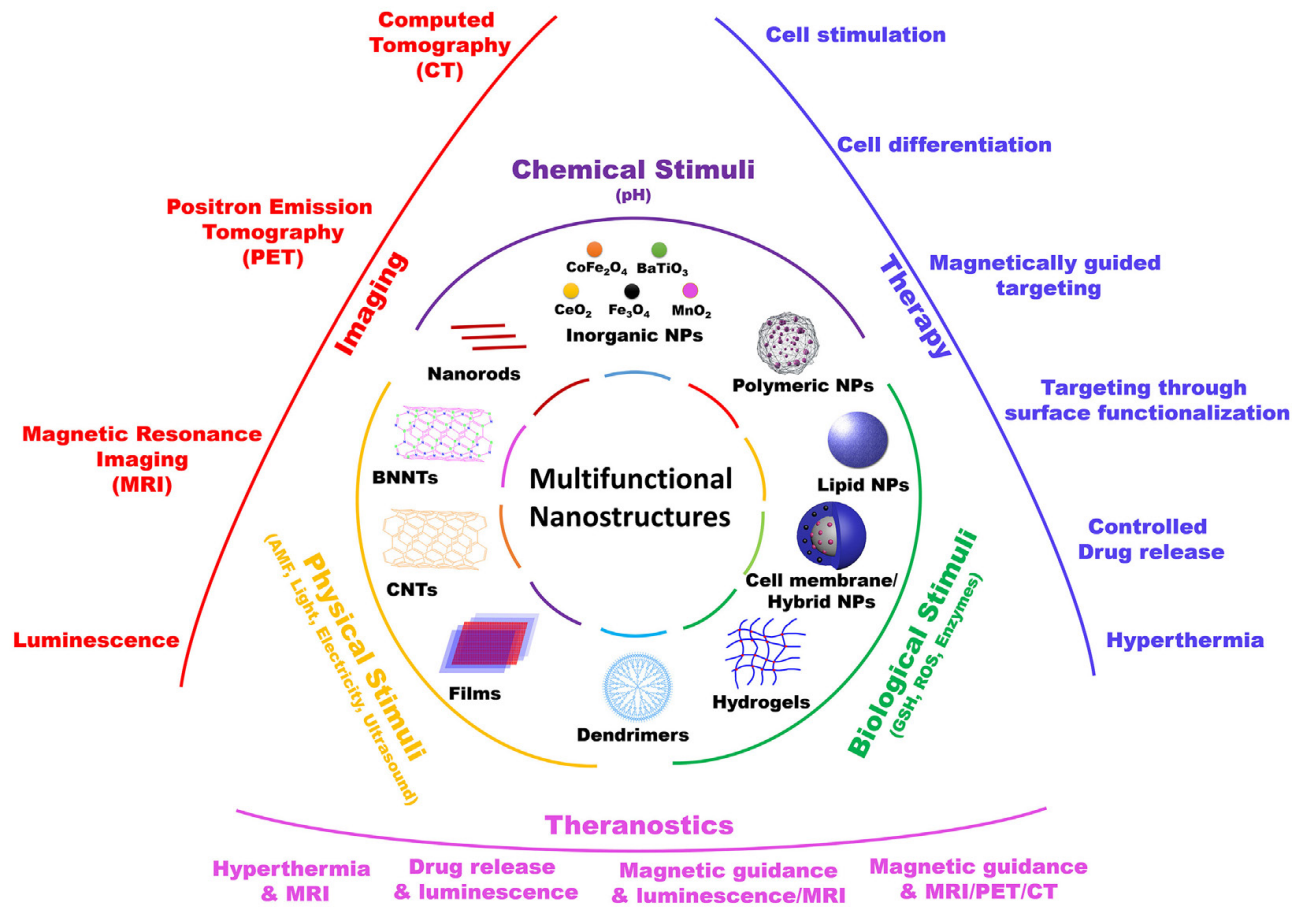
Schematic representation of various multifunctional nanostructures, their responsive stimuli, and their main biomedical applications.

In this mini-review, we will focus on those nanomaterials that, in our opinion, are the most promising in terms of clinical translation, with particular attention to nanoparticles that act as “nano-transducers,” allowing for a remote manipulation of biological activities, and thus providing a “smart” interface between biological and non-biological environments (Figure 1).

## Remote Cell Stimulation Through Smart Nanomaterials

The possibility to finely and remotely manipulate cell behavior in deep tissues is of extreme importance in medicine for restoring physiological cell activities after the onset of a pathological condition (Dell’Anno et al., 2014; Li et al., 2017). Furthermore, the remote control of cell activities *in vivo* allows the elucidation of mechanisms at the base of different diseases and the development of novel therapeutic strategies (Brunoni et al., 2012; Paz et al., 2013; Legon et al., 2014).

A consolidated method for the fine modulation of the activity of specific cell types is represented by optogenetics, which consists in the genetic sensitization of targeted cells to light through a promoter-driven expression of light-sensitive proteins. Alternatively, a new generation of smart nanomaterial-based approaches for the remote control of cell behavior has recently been proposed (Genchi et al., 2017a). Smart nanomaterials can be externally/wirelessly activated by different energy sources [e.g., near-infrared (NIR) radiations, radiofrequency stimulations, magnetic fields, ultrasounds, etc.] that are able to penetrate biological tissues efficiently and non-invasively. Nanostructure activation in deep tissues triggers specific behaviors (e.g., neural spikes and myocyte contractions) (Eom et al., 2014; Colombo et al., 2016; Marino et al., 2017a), or tunes biochemical pathways involved in different cell activities, such as differentiation (Kim et al., 2016; Rau et al., 2016), morphological maturation (Ciofani et al., 2010), and hormone release (Stanley et al., 2012, 2015). These energy-driven nanoparticle-mediated approaches are able to overcome the scarce tissue penetration by visible light and the use of viruses to genetically modify target cells, which are the main drawbacks currently limiting clinical applications of optogenetics (Jarvis and Schultz, 2015).

Piezoelectric nanomaterials are a class of nanostructures able to generate a voltage on their surface when exposed to a mechanical stimulation, for example by means of ultrasounds, US (Wang et al., 2007). This voltage has been used for the stimulation of electrically excitable cells, like neurons (Ciofani et al., 2010; Royo-Gascon et al., 2013; Hoop et al., 2017; Lee et al., 2017) and bone cells (Genchi et al., 2017b). Our group demonstrated for the first time that the acute US-driven piezo-stimulation of barium titanate nanoparticles (BTNPs) associated to plasma membrane was able to significantly increase the intracellular calcium concentration in neural cells (Marino et al., 2015). The combination of US and non-piezoelectric BTNPs was not able to elicit a significant neural response, thus confirming that the mechanism was mediated by piezoelectricity and not by other unspecific phenomena (e.g., mechanical or thermal).

Another wide-spread approach for remote cell activation is represented by nanoparticle-assisted heat stimulation by short-duration temperature increments in a physiological range (of about 5°C) (Shapiro et al., 2012). A local increment of temperature can be obtained by exploiting different energy transduction approaches, such as the photothermal and the magnetothermal ones. Photothermal stimulation consists in the transduction of photon energy into heat and can be remotely triggered with NIR radiation in combination with many plasmonic nanomaterials, such as gold nanoshells (Erickson and Tunnell, 2010), gold nanorods (Huang et al., 2006), single-walled carbon nanotubes (Mocan et al., 2011), graphene oxide (Robinson et al., 2011), and copper sulfide (Cu_2_S) nanocrystals (Wang et al., 2015). Different independent works showed that photothermal stimulation is able to reversibly elicit a neural response in terms of spike activity, intracellular calcium levels, and neurite outgrowth (Yong et al., 2014; Paviolo and Stoddart, 2017). These effects seem to be mediated by the opening of temperature-sensitive calcium channels (Miyako et al., 2014) and/or by heat-dependent capacitance changes of the neural plasma membrane (Carvalho-de-Souza et al., 2015).

However, the mechanisms of photothermal stimulation on complex neural networks have to be further investigated. Indeed, a recent work documented an inhibited neural network activity of hippocampal primary culture treated with gold nanorods upon NIR irradiation (Yoo et al., 2014). Remote photothermal nerve activation was successfully used for inducing leg muscle contraction in frogs after treatment with carbon nanohorns and NIR irradiation (Miyako et al., 2014). Similarly to neural cells, muscle cells can be stimulated by heat: in this concern, our group has recently demonstrated that an acute NIR irradiation of gold nanoshell-containing cultures is able to induce myotube contraction, while a chronic one suggested to promote mitochondriogenesis (Marino et al., 2017a).

Alternatively to photothermal stimulation, magnetothermal transduction can be exploited for cell heating/stimulation. In this case, magnetic nanoparticles dissipate heat when undergo an alternate electric field (Noh et al., 2017). In a recent work, this approach was exploited for remote deep stimulation of the ventral tegmental area (VTA) through the opening of a transfected heat-sensitive receptor TRPV1 in mice. The consequent increase of neural activity was also observed in the brain areas receiving excitatory projections from VTA. Interestingly, the retention period of the magnetic nanoparticles in the VTA was longer than a month, thus allowing for chronic magnetothermal VTA stimulations (Chen et al., 2015).

Other nanomaterial-assisted remote stimulation methods exploit magnetoelectric and optoelectric phenomena (Colombo et al., 2016; Wang and Guo, 2016). Concerning magnetoelectric materials, cobalt ferrite–barium titanate (CoFe_2_O_4_–BaTiO_3_) core-shell nanoparticles were used to modulate deep-brain activity under a low-intensity alternating magnetic field (Guduru et al., 2015). Moreover, the magnetic properties of these nanoparticles also facilitated their delivery to the central nervous system (CNS). Indeed, CoFe_2_O_4_–BaTiO_3_ nanoparticles were intravenously administered in mouse tail and forced to cross the blood-brain barrier (BBB) with a static magnetic field.

Regarding the frontiers of optoelectronic bio-interfaces, polymeric biocompatible organic nanoparticles/films of poly(3-hexylthiophene) revealed great performances both in restoring the functionality of blind retinas on *ex vivo* models [i.e., explants of rat retinas characterized by photoreceptor degeneration (Ghezzi et al., 2013)], and in modulating the behavior of eyeless animals (i.e., freshwater polyps) by amplifying the function of their primitive photoreceptors (Tortiglione et al., 2017).

As a further example, a class of nanomaterials characterized by elements with high atomic number *Z* (e.g., Au/Pt nanoparticles) can be also exploited as contrast agents and sensitizers of X-rays, representing very promising nanovectors for cancer theranostics (Subiel et al., 2016). The great versatility and potential of these nanomaterials in nanomedicine is noteworthy, especially considering their great potential also in proton therapy (Schlatholter et al., 2016).

## Multifunctional Drug Delivery Systems (MDDS)

The optimization of carriers for delivering drugs specifically to diseased areas originated from the need to overcome drug limitations, such as cytotoxicity, immunogenicity, short circulation times, non-controlled bio-distribution, and the non-targeting ability toward specific tissues. During recent years, significant advancements in the field of nanobiotechnology led to the development of MDDS for the accomplishment of diagnostic and therapeutic purposes through a single medical device, able of performing bio-imaging duties, while improving the therapeutic efficacy of drugs available on the market. MDDS already presented in the literature can have a variety of morphologies and sizes, and can be made of various materials like natural and synthetic polymers, lipids, and other inorganic and organic materials. Nanostructures made of polymeric materials are the most used MDDS, due to a number of advantages, including the high versatility during their fabrication, their controllable size and shape, their high encapsulation efficiency, and their easy surface functionalization by numerous targeting groups, including peptides, proteins, and antibodies. Furthermore, coating of these nanostructures with stealth materials like poly(ethylene glycol), PEG, increases their circulation time inside the body. In combination with the targeting ability, this renders them suitable for the specific delivery of a number of therapeutic preparations, including drugs, enzymes, proteins, RNA, and DNA (Mura et al., 2013; Srinivasan et al., 2015; Bose et al., 2016; Lu et al., 2016) to specific diseased tissues. The most used polymeric nanostructures are nanoparticles, nanocapsules, dendrimers, nanospheres, spherical and worm-like micelles, nanotubes, and hydrogels (Theato et al., 2013; Torchilin, 2014; Lu et al., 2016).

Another category of nanomaterials used as MDDS is represented by lipid nanostructures, including niosomes, transfersomes, liposomes, solid lipid nanoparticles, and nanostructured lipid carriers. Lipid-based nanostructures are considered a better alternative compared to polymeric nanostructures, especially for the treatment of CNS diseases, due to their inherent biocompatibility, low immunogenicity, and their ability to penetrate the BBB. A newer approach, similar to that of lipid-based nanostructures, but more biomimetic, relies on cell membrane-derived vesicles or the coating of existing nanoparticles such as poly(lactide-co-glycolide) and magnetite nanoparticles with the cell membrane of various cells, like macrophages, neutrophils, red blood cells, cancer cells, and others (Fang et al., 2014; Luk and Zhang, 2015; Bose et al., 2016; Gao et al., 2016; Krishnamurthy et al., 2016).

Inorganic nano- and microparticles, including magnetite (Fe_3_O_4_), maghemite (γ-Fe_2_O_3_), manganese dioxide (MnO_2_), cerium dioxide (CeO_2_), platinum (Pt), silver (Ag), zinc oxide (ZnO_2_), silica (SiO_2_), titanium dioxide (TiO_2_), and others, have also been used as MDDS combining various functionalities, such as imaging and hyperthermia (Hayashi et al., 2013; Tapeinos et al., 2016), reactive oxygen species (ROS) scavenging and oxygen generation (Bizeau et al., 2017; Tapeinos et al., 2017), and antimicrobial and anticancer activities (Mohanta et al., 2017).

A combination of the above-mentioned structures like polymers and lipids, polymers and inorganic nanoparticles, and lipids and inorganic nanoparticles have also been studied aiming at further improving the multifunctionality and the therapeutic effect of these nanostructures. Our group, for instance, used a lipid matrix to encapsulate iron oxide nanoparticles and the drug sorafenib, and subsequently showed its magnetically-driven accumulation *in vitro* against hepatocarcinoma cells, demonstrating localized therapy (Grillone et al., 2015). A combination of inorganic and polymeric nanoparticles have also been used for multimodal imaging of breast cancer tissue (Cheng et al., 2012), providing a more precise imaging through luminescence and magnetic resonance.

The multifunctionality of each one of the above-mentioned nanostructures derives from the combination of materials with specific characteristics and can be translated as the response of the MDDS to a physical/external and/or a biological/internal stimulus, by changing their morphological (size, shape) and/or their physicochemical characteristics (colloidal stability, crystal structure, hydrophobicity, redox state, etc.). To date, numerous systems that respond to external and/or internal stimuli have been developed, but the most important are those ones that respond to more than one stimulus and show ability of a most precise control in the release of the encapsulated therapeutic molecules. Stimuli–responsive systems are usually a combination of polymeric nanostructures and inorganic nanoparticles (Tapeinos et al., 2013, 2016, 2017), although lipid-based nanostructures have also been developed to alter their properties when an external stimulus is applied (Du et al., 2015).

Stimuli-responsive nanostructures that have been used for the treatment of various diseases can respond to physical stimuli, including ultrasounds, light, electric fields, and magnetic fields (Mura et al., 2013; Marino et al., 2015, 2017b,c; Genchi et al., 2016, 2017b), or they can take advantage of the changes in the biological microenvironment of each disease and respond to alterations in temperature, pH, redox conditions, ROS, and enzyme concentration (Tapeinos et al., 2008, 2017; de la Rica et al., 2012; Torchilin, 2014; Tapeinos and Pandit, 2016; Bizeau et al., 2017). Furthermore, a combination of these stimuli (Mura et al., 2013; Tapeinos et al., 2013, 2016; Efthimiadou et al., 2014a,b; Lu et al., 2016) have also been used to increase the therapeutic versatility of the MDDS.

## Applications and Future Perspectives

An increasing number of nanotechnology-based strategies are available for wireless and low-invasiveness manipulation of biological activities in hardly accessible anatomical districts, as the CNS. Among them, ultrasound activation of piezoelectric materials is highly promising for the treatment of deep tissues (Tufail et al., 2011). Further investigations are, however, necessary for selective delivery of piezoelectric nanomaterials to target tissues and retention on site *in vivo*. The first studies on toxicity and accumulation of piezoelectric boron nitride nanotubes was conducted by our group with rabbits. Our studies collectively demonstrated high biocompatibility in terms of liver, kidney, and blood parameters of high doses (up to 10 mg/kg) of intravenously injected nanotubes (Ciofani et al., 2012, 2013a).

Our group also provided the first evidences on the applicability of piezoelectric nanocomposite films based on poly(vinylidenefluoride-co-trifluoroethylene), P(VDF-TrFE), and BTNPs to neuron-like cell stimulation for cochlear prosthetics. Aiming at compensating missing/altered hair cell function, our nanocomposite films were satisfactorily tested for stimulation of a human neuronal model by direct piezoelectric effect (Genchi et al., 2016). A single US application to films supporting SH-SY5Y cell cultures triggered significantly higher calcium influxes than plain P(VDF-TrFE) films and non-piezoelectric control substrates. Repeated stimulations significantly increased expression of β3-tubulin and neurite extension due to the better piezoelectric properties of composite films, suggesting improved functional maturation of the neuronal model on our artificial cochlear epithelium.

Smart nanomaterials are providing concrete opportunities for the treatment of pathological conditions that affect deep anatomical districts. However, hybrid smart material devices are opening even unprecedented therapeutic opportunities in nanomedicine, like biological barrier overcoming while multiple functions are accomplished (imaging, drug release, etc.). In particular, low-intensity magnetic field stimulation of magnetoelectric nanomaterials holds promise of clinical practice application in the near future. For instance, CoFe_2_O_4_–BaTiO_3_ were validated for Parkinson’s disease treatment *in silico* (Yue et al., 2012). Moreover, they were successfully tested for magnetically driven BBB crossing, as well as for coupling with and mapping the intrinsic neural activity in mice (Guduru et al., 2015). CoFe_2_O_4_–BaTiO_3_ nanoparticles were also used for direct current-field cell targeting and alternating current-field releasing of anti-HIV drug *in vitro* (Nair et al., 2013) and of paclitaxel for ovarian cancer treatment *in vivo* (Rodzinski et al., 2016).

Highly encouraging to the treatment of deep tissues are also cerium dioxide nanomaterials, alone and in combinations with other materials for the control of redox environments (both intercellular and intracellular ones). Our group demonstrated suitability of CeO_2_ nanoparticles to ROS scavenging *in vitro* and *in vivo*, with relevant findings to Parkinson’s disease (Ciofani et al., 2013b, 2014) and obesity treatment (Rocca et al., 2014, 2015). However, the great potentialities of nanoceria can find full exploitation by targeting specific cell populations and intracellular anatomic districts. A first example of multifunctional platform based on CeO_2_ nanoparticles involved coating with β-cyclodextrin and association to ferrocene-mesoporous SiO_2_ nanoparticles, MSN (Xu et al., 2013). These hybrid nanoparticles promoted HEK293 normal cell proliferation, while they limited that one of A549 cancer cells. Moreover, they mostly localized in the cytosol of HEK293, whereas in the lysosomes of A549. In these acidic organelles, the nanoparticles underwent dissolution and exerted a strong cytotoxic effect by the synergic action of CeO_2_ nanoparticles and oxidized ferrocene. This anticancer activity was even increased when the MSN pores were loaded with floxuridine as a antitumor drug model. In this way, CeO_2_-based nanoparticles accomplished at the same time intracellular compartment targeting, antitumoral activity, and drug delivery functions.

Other interesting multifunctional materials responsive to external stimuli are halloysite nanotubes (HNTs), that for instance were used for the preparation of temperature-responsive platforms by poly(*N*-isopropylacrylamide) coating and curcumin loading, for subsequent release upon temperature increase (Cavallaro et al., 2015). In particular, the platforms were tested in acidic environment simulating the gastrointestinal transit and exerted successful protection/delivery of their curcumin payload with promising implications in cancer treatment. In another study, HNTs were coated with folate *via* a complex redox-responsive disulfide bond and used for targeting/release of doxorubicin in a reducing environment *in vitro*, also providing safe drug delivery to tumor sites in nude mice (Hu et al., 2017).

A detailed list of multifunctional nanostructures, their responsive stimuli, and their biological effects can be found in Table 1.

**Table 1:** Stimuli and effects of various multifunctional responsive structures.

Responsive material	Stimulus	Effect	Reference
Au nanorods	Photothermal (NIR radiation)	Neuronal spikes	Eom et al. (2014)
Au@SiO_2_ nanoshells	Photothermal (NIR radiation)	Myotube contractions	Marino et al. (2017a)
Peptide ligands assembled on carbon nanotubes	Light	Cell differentiation	Kim et al. (2016)
Au nanoparticles	Photothermal	Cell differentiation	Rau et al. (2016)
Cu_2_S nanocrystals	Photothermal and photodynamic (NIR radiation)	Hyperthermia and ROS-induced apoptosis	Wang et al. (2015)
Au nanorods	Photothermal (NIR radiation)	Cancer cell imaging and photothermal therapy	Huang et al. (2006)
Au nanorods	Photothermal (NIR radiation)	Inhibition of spontaneous and epileptiform neural activity	Yoo et al. (2014)
Au nanorods	Photothermal (NIR radiation)	Evoking spikes on primary auditory neurons	Yong et al. (2014)
Carbon nanohorns	Photothermal (NIR radiation)	Nerve activation (opening of the temperature-sensitive calcium channels)	Miyako et al. (2014)
Ultrasmall reduced graphene oxide	NIR radiation	Photoablation of U87MG cancer cells	Robinson et al. (2011)
ZnO nanowires	Ultrasounds	Continuous direct-current output	Wang et al. (2007)
BNNTs	Ultrasounds	Neural stimulation (neurite outgrowth)	Ciofani et al. (2010)
BaTiO_3_ nanoparticles with tetragonal crystal	Ultrasounds	Neural stimulation (calcium and sodium waves)	Marino et al. (2015)
PVDF film	Mechanical vibration	Neural stimulation (neurite outgrowth)	Royo-Gascon et al. (2013)
PVDF membranes	Ultrasounds	Neural differentiation	Hoop et al. (2017)
P(VDF-TrFE) conduits	N/A	Regeneration of transected adult rat spinal cord	Lee et al. (2017)
P(VDF-TrFE)/BTNP composite films	Ultrasounds	Stimulation of a human neuronal model Increased calcium influx Increased expression of β3-tubulin Neurite extension	Genchi et al. (2016)
P(VDF-TrFE)/BNNT composite films	Ultrasounds	Osteogenic differentiation	Genchi et al. (2017b)
High-Z nanomaterials	Ionizing radiations	Enhancement of irradiation effect	Schlatholter et al. (2016); Subiel et al. (2016)
Fe_3_O_4_ magnetic nanoparticles	Magnetic field	Deep stimulation of the ventral tegmental area through opening of the transfected heat-sensitive receptor TRPV1	Chen et al. (2015)
CeO_2_	ROS concentration	ROS scavenging	Ciofani et al. (2013b, 2014); Rocca et al. (2014, 2015)
CoFe_2_O_4_-BaTiO_3_	Ultrasounds/static magnetic field	Magnetically guided targeting	Yue et al. (2012)
	Direct current-field	Cell targeting	Nair et al. (2013); Rodzinski et al. (2016)
	Alternating current-field	Drug release	
Fe_3_O_4_, γ-Fe_2_O_3_	Alternating magnetic field	Magnetic resonance imaging Controlled release Tumor reduction	Hayashi et al. (2013); Tapeinos et al. (2016)
MnO_2_	ROS concentration	ROS scavenging and oxygen generation	Bizeau et al. (2017); Tapeinos et al. (2017)
CoFe_2_O_4_-BaTiO_3_	Low-intensity alternating magnetic field/static magnetic field	Modulation of deep-brain activity/guided brain targeting	Guduru et al. (2015)
Poly(3-hexylthiophene)	Light	Restoration of the functionality of blind retinas	Ghezzi et al. (2013)
		Modulation of the behavior of eyeless animals	Tortiglione et al. (2017)
β-cyclodextrin/CeO_2_	ROS concentration	Intracellular compartment targeting, enhanced antitumoral activity and drug delivery	Xu et al. (2013)
Ferrocene/SiO_2_			
Poly (acrylic acid), Fe_3_O_4_, Au, NaYF_4_: Yb, Er	Magnetic field NIR irradiation	Multimodal imaging of breast cancer tissue Magnetically targeted photothermal therapy	Cheng et al. (2012)
Cetyl palmitate/Fe_3_O_4_	Static magnetic field	Localized anticancer therapy	Grillone et al. (2015)
DPPC, DSPE-PEG_2000_-folate, C60-Fe_3_O_4_-PEG_2000_	Radiofrequency	Magnetic resonance imaging Photothermal ablation Controlled release	Du et al. (2015)
DMAEMA, AA, Disulfide, Fe_3_O_4_	Alternating magnetic field	Enhanced release of encapsulated anticancer drugs	Tapeinos et al. (2013, 2016)
	Temperature		
	pH		
	GSH concentration		
Carbon-based	NIR irradiation	Stem-cell differentiation	Kim et al. (2016)
	Laser Irradiation	Photothermal ablation	Mocan et al. (2011)
Cell membrane of various cells, like macrophages, neutrophils, red blood cells, cancer cells	N/A	Tumor-specific immune response Specific targeting Drug delivery	Fang et al. (2014); Luk and Zhang (2015); Bose et al. (2016); Gao et al. (2016); Krishnamurthy et al. (2016)
Iron oxide nanoparticles and genetically encoded ferritine nanoparticles	Radiowave heating	Insulin transgene expression and proinsulin release	Stanley et al. (2012, 2015)
Au nanoparticles	Photothermal (light at 532 nm)	Evoking spikes to hippocampal neurons through heat-dependent changes of capacitance of the neural plasma membrane	Carvalho-de-Souza et al. (2015)

*AA, acrylic acid; BNNTs, boron nitride nanotubes; BTNPs, barium titanate nanoparticles; C60, fullerene; Disulfide, *N,N*′-(disulfanediylbis (ethane-2,1-diyl))bis(2-methylacrylamide); DMAEMA, dimethyl aminoethyl methacrylate; DPPC, dipalmitoylphosphatidylcholine; DSPE-PEG_2000_-folate, 1,2-distearoyl-sn-glycero-3-phospho-ethanolamine-*N*-[carboxy (poly(ethylene glycol))-2000]-folate; GSH, glutathione; NIR, near infrared; PEG, poly(ethylene glycol); PVDF, poly(vinylidenefluoride); P(VDF-TrFE), poly(vinylidenefluoride-co-trifluoroethylene); ROS, reactive oxygen species*.

## Conclusion

To date, numerous studies have reported the enhanced therapeutic effect of several nanostructured devices compared to traditional systems. However, a lot of work is still mandatory for these materials to provide a solution to unmet clinical needs. Non-invasive approaches for remote targeting and activation are nowadays available, yet the development of complex and reliable models of biological barriers *in vitro* and *in vivo* is necessary to ensure proper device testing. In our opinion, the enhancement of the capability of intracellular compartment targeting by fine tuning/control of the surface physicochemical properties represents a key step for these smart materials to be made available to the next future clinical practice.

## Author Contributions

GC wrote the Section “Introduction,” GG wrote the Sections “Applications and Future Perspectives” and “Conclusion,” AM wrote the “Remote cell stimulation through smart nanoparticles” Section, and CT wrote the “Multifunctional drug delivery systems” Section and prepared Figure and Table. GC revised the whole manuscript.

## Conflict of Interest Statement

The authors declare that the research was conducted in the absence of any commercial or financial relationships that could be construed as a potential conflict of interest.
